# Prognosis value of RBBP8 expression in plasma cell myeloma

**DOI:** 10.1038/s41417-018-0069-3

**Published:** 2019-01-09

**Authors:** Weilong Zhang, Ying Song, Xue He, Xiaoni Liu, Ye Zhang, Zuozhen Yang, Ping Yang, Jing Wang, Kai Hu, Weiyou Liu, Xiuru Zhang, Xiaoliang Yuan, Hongmei Jing

**Affiliations:** 1 Department of Hematology, Lymphoma Research Center, Peking University Third Hospital, Beijing, 100191 China; 2 Gannan Medical University, Ganzhou, 341000 China; 3 Department of Pathology, Beijing Tiantan Hospital Affiliated with Capital Medical University, No. 6 Tiantan Xili, Beijing, 100050 China; 4 Department of Respiratory Medicine, The First Affiliated Hospital of Gannan Medical University, Ganzhou, 341000 China; 5 Melbourne School of Population and Global Health, The University of Melbourne, Victoria, 3010 Australia

**Keywords:** Myeloma, Cancer genetics

## Abstract

Plasma cell myeloma (PCM) secretes monoclonal immunoglobulin (Ig) by clonal plasma cells of abnormal proliferation in the bone marrow. As PCM is incurable, it is necessary to find new biomarkers to predict the prognosis and recurrence of PCM. The relationship between cancer and RBBP8 has not been fully studied. The role of RBBP8 in tumorigenesis remains inconsistent. We described the expression of RBBP8 in the gene expression profile of 1930 PCM samples (1878 PCM patients) from seven independent data sets. We analyzed the relationship between RBBP8 and survival prognosis, recurrence, and treatment response in patients with PCM, and the biological significance of RBBP8 in PCM. The gene expression level of RBBP8 was significantly related to the International staging system (ISS) grade of PCM (*P* = 0.0012). RBBP8 expression in different molecular subtypes was different (*P* < 2.2e-16). High RBBP8 expression is associated with poor survival in PCM (*P* < 0.0001). High expression of RBBP8 indicates that PCM patients are more likely to relapse (*P* = 0.0078). The biological significance of RBBP8 in PCM is related to the cell cycle (*P* < 0.05). High RBBP8 expression predicts poorer survival and more likely relapse in PCM. RBBP8 plays an important role in the cell cycle of PCM. RBBP8 can be considered an independent prognostic factor for PCM. RBBP8 can be used as a potential biomarker for assessing the prognosis of PCM patients.

## Introduction

Plasma cell myeloma (PCM) secretes monoclonal immunoglobulin (Ig) by clonal plasma cells of abnormal proliferation in the bone marrow. Clinical features of PCM typically present with bone damage, hypercalcemia, renal impairment, and anemia. PCM accounts for ~ 10% of cancers in the blood system [1]. The average median survival of the disease is 3–4 years. Costa LJ summarized that the early mortality was 4–25% of newly diagnosed PCM patients in a randomized phase 3 clinical trial in the past decade [2]. However, he found that the early mortality rate is more higher than what has been reported in clinical trials [2]. At present, the treatment of novel myeloma drugs of immunomodulatory agents and proteasome inhibitors improve survival rates [3, 4]. In addition, combinations of high dose-chemotherapy with autologous stem cell transplantation (ASCT) resulted in better overall survival (OS) [5–7]. However, there is still a high recurrence rate in PCM. Therefore, it is necessary to find new biomarkers to predict the prognosis and recurrence of PCM.

According to the genetic classification, PCM can be classified based on the translocation and cyclin D (TC) and the University of Arkansas for Medical Sciences (UAMS) system. The TC classification distinguishes eight subgroups by the deactivation of primary immunoglobulin H translocations and transcriptional activation of cyclin D gene [8]. The UAMS molecular classification were classified into seven subtypes by different gene expression profiles, including MMSET [t(4;14)], MAF [t(14;16)/t(14;20)], CD1/2 [t(11;14), and t(6;14)], HY (hyper diploid cluster), PR (proliferation), and a cluster mainly characterized by a low percentage of bone disease (LB) [9]. Based on the UAMS classification in 2010, PCM is reclassified as CD1, CD2, CTA, HY, MF, MS, myeloid, NFKB, and PR [10]. According to treatment response with bortezomib and dexamethasone (Dex), patients were divided into CR (complete response) group, PR (partial response) group, MR (minimum response) group, NC (no change) group, and PD (progressive disease) group [11]. Similarly, according to the treatment response with after induction chemotherapy (pre-1st) and after ASCT, patients were classified into CR group, VGPR (very good partial response) group, PR group, NR (stable disease) group, and Prog (no response, progressive disease) group [12].

The human RBBP8 (retinoblastoma-binding protein 8), also known as CTIP (CTBP (C-terminal-binding protein)-interacting protein), is a protein coding gene. This protein interacts with other factors and participates in a variety of nuclear pathways. Overexpression of RBBP8 in tumors is mainly related to cyclin D1 transcription [13]. CTIP/RBBP8 gene accelerates tumorigenesis through transcriptional activity [14]. CTIP/RBBP8 was described as a key checkpoint of G_1_ phase by initiation S-phase and DNA replication [15]. CTIP/RBBP8 mediates DNA double-strand breaks repair in the cell cycle through homologous recombination [16, 17]. CTIP/RBBP8 interacts with proliferating cell nuclear antigen (PCNA) at specific localization and activates DNA damage checkpoints leading to DNA damage, suppressing DNA replication at S and G2 phases [18]. However, the relationship between cancer and RBBP8 has not been fully studied.

Investigation found RBBP8 genes was associated with sporadic brain arteriovenous malformations [19]. Advanced invasive bladder cancer was associated with the deletion of nuclear RBBP8 protein [20]. Deletion of the RBBP8 gene was associated with significantly worse prognosis in ovarian cancer [21]. The poor prognosis of breast cancer was related to the low or no expression of RBBP8 [22, 23]. RBBP8 is also overexpressed in certain tumors [24]. However, despite these associations with cancer, the expression level of RBBP8 has not been reported in PCM. We analyzed the association of RBBP8 expression with PCM prognosis, relapse, and event-free survival (EFS) or OS.

## Materials and methods

### Data source and gene expression analysis

Probe set measurements for all arrays were calculated using the RMA (robust multiarray averaging) method. Logarithmic conversion of relative RNA expression values was performed using log2. According to the RBBP8 gene expression level, RBBP8-high group, and RBBP8-low group using survminer package with maximally selected rank statistics arithmetic. Only genes with foldchanges (log2) > 0.8 or < −0.8 were considered different expressed genes. *P* values < 0.05 were defined to be statistically significant. We obtained seven independent data set totally 1930 PCM samples (1878 PCM patients) from the seven independent Gene Expression Omnibus (GEO) data sets. This study was in accordance with the Helsinki Declaration.

GSE24080 of 559 patients were obtained from the GEO database. The gene expression was detected by Affymetrix Human Genome U133 Plus 2.0 Array [25]. We analyzed the relationship between RBBP8 expression and International staging system (ISS) stage, 1q21 amplification, molecular subtype, and survival.

GSE19784 of 311 patients were obtained from the GEO database. The gene expression was detected by using Affymetrix GeneChip U133 plus 2.0 arrays [10]. Hierarchical clustering identified 10 distinct subgroups. We analyzed the relationship between RBBP8 expression and molecular subtype (9 subgroups).

GSE9782 of 477 patients were obtained from the GEO database. Gene expression profiling was detected using the Affymetrix 133 A/B microarray [11]. We analyzed RBBP8 expression in different therapeutic response with bortezomib or dexamethasone (Dex).

GSE83503 of 585 patients were obtained from the GEO database. The gene expression array was Affymetrix Human Exon 1.0 ST Array [26]. We analyzed the relationship between RBBP8 and PCM recurrence.

GSE82307 of 66 samples (33 patients) were obtained from the GEO database. Samples were tested by Affymetrix Human Genome U133 Plus 2.0 Array [27]. We analyzed the relationship between RBBP8 expression in presentation (baseline) and recurrence.

GSE19554 of 38 samples (19 patients) were obtained from the GEO database. Samples were tested by Affymetrix Human Genome U133 Plus 2.0 Array [28]. We analyzed the relationship between RBBP8 expression at diagnosis (baseline) and after induction chemotherapy (pre-1st).

GSE39754 of 136 patients were obtained from the GEO database. The gene expression was detected by Affymetrix Human Exon 1.0 ST Array [12]. We analyzed RBBP8 expression in different therapeutic response with the pre-1st and after ASCT.

### Gene ontology (GO) analysis

Pathway enrichment was performed using the DAVID tool with default parameters to analyze different expression genes between RBBP8-high groups and RBBP8-low groups in PCM (from data set GSE24080). GO pathway analysis results are showed in Fig. 4b.

### Statistics

This study used R software v3.1.3 (ggplot2 and survminer package) for statistical analysis. Kruskal–Wallis test was used for comparison of RBBP8 expression levels between ISS grades. RBBP8 expression levels were compared between different molecular typing groups and different treatment response groups using one-way analysis of variance. Kaplan–Meier curves, log-rank test, Cox regression are used in survival analysis. RBBP8 expression comparison of 33 pairs specimens before and after recurrence and 19 pairs specimens before and after treatment using Wilcoxon test. For all statistical methods, the *P* < 0.05 was considered to indicate statistical significance.

## Results

### Expression level of RBBP8 in different molecular subtypes of PCM

**Fig. 1:**
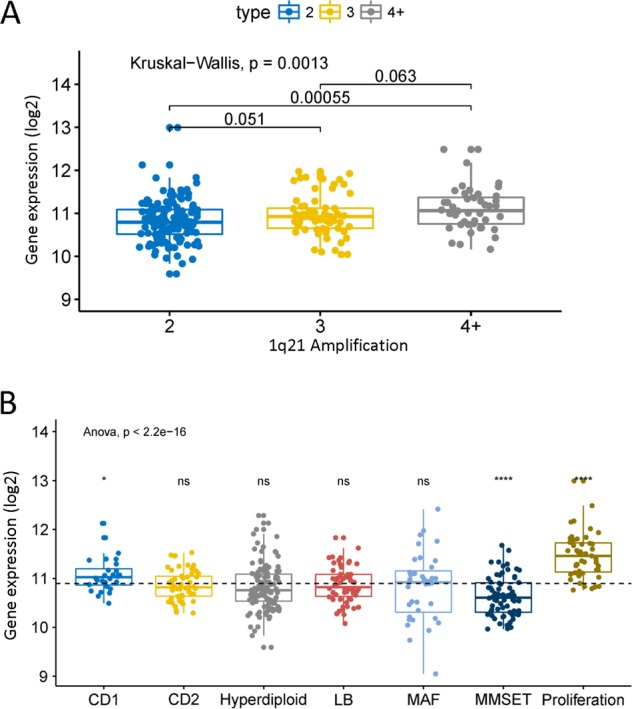
RBBP8 gene expression in different molecular types of PCM. **a** Comparison of RBBP8 expression levels at different amplification levels of 1q21, The *X* axis represents the 1q21 amplification level and the *Y* axis represents gene expression. RBBP8 gene expression was measured as log2. *P* = 0.0013, Kruskal–Wallis test. **b** Comparison of RBBP8 expression levels in PCM of seven molecular subtypes. The *X* axis represents different molecular subtypes and the *Y* axis represents gene expression (log2). *P* < 2.2e-16, ANOVA test, ns, * and **** indicate *P* > 0.05, *P* ≤ 0.05, and *P*  ≤ 0.0001, respectively. The average of the entire data is used as a reference group. Each group level is compared with the reference group. Add a horizontal dashed line at the average of reference group

The amplification of 1q21 was related to the expression level of RBBP8 (Fig. 1a, *P* = 0.0013, Kruskal–Wallis test). Compared with the two copies of 1q21 PCM samples, RBBP8 was significantly increased in the 1q21 amplification samples ( ≥ 4 copies) (Fig. 1a, *P* = 0.00055, Wilcoxon test). The expression of RBBP8 in different molecular subtypes was different (Fig. 1b, *P* < 2.2e-16, one-way analysis of variance analysis test). The expressions of RBBP8 in CD1 and PR subtypes were increasing, compared with the mean of all subtypes (Fig. 1b, CD1, *P* ≤ 0.05; PR, *P* ≤ 0.0001, unpaired *t* test, two sided). Although MMSET subtype showed the lower RBBP8 expressions (Fig. 1b, *P* < = 0.0001, unpaired *t* test, two sided), and CD2, HY, LB, and MAF subtypes were no significantly difference (Fig. 1b, *P* > 0.05, unpaired *t* test, two sided). In another molecular subtype classification, the expression of RBBP8 was also different (Fig. S2, *P* < 2.2e-16, one-way analysis of variance analysis test). Compared with the average of all subtypes, CD1, CD2, MF, and PR subtypes were showing higher RBBP8 expressions in GSE19784 data set (Fig. S2, CD1, *P* ≤ 0.05; CD2, *P* ≤ 0.01; MF, *P* ≤ 0.01; PR, *P* ≤ 0.0001, unpaired *t* test, two sided). Although CTA and MMSET subtypes were showing the lower RBBP8 expressions (Fig. S2, *P* ≤ 0.0001 unpaired *t* test, two sided), and HY, MS, and myeloid subtypes were no significantly difference (Fig. S2, *P* > 0.05, unpaired *t* test, two sided).

### Expression level of RBBP8 in different ISS stages of PCM

To investigate the expression pattern of RBBP8 in PCM, we analyzed expression profile of PCM from data set GSE24080. The gene expression level of RBBP8 was significantly related to the ISS grade of PCM (Fig. S1A, *P* = 0.0012, Kruskal–Wallis test). Compared with ISS grade I PCM, ISS grade II and grade III PCM showed higher expression of RBBP8 (Fig. S1A, *P* = 0.0019, *P* = 0.0049, Wilcoxon test). This result indicated that high expression of RBBP8 was a sign of severity of PCM. We also studied the expression levels of RBBP8 in different ISS grades among different serotypes of PCM. According to the serum immunoglobulin component, PCM can be divided into eight types. We listed three common serotypes FLC (free light chain), IgA and IgG serotypes. We founded that there was a significant difference in the expression of RBBP8 among different ISS grades of IgG serotypes (Fig. S1B, *P* = 6.6e-05, Kruskal–Wallis test). With the increase of ISS grades, the expression of RBBP8 gene was upregulated (Fig. S1B, *P* = 0.019, *P* = 2.8e-05, *P* = 0.058, Wilcoxon test). The expression of RBBP8 was no significant difference in the different ISS grades of the IgA and FLC serotypes (Fig. S1B, IgA, *P* = 0.17; FLC, *P* = 0.16, Kruskal–Wallis test).

### Expression levels of RBBP8 was associated with relapse in PCM

**Fig. 2:**
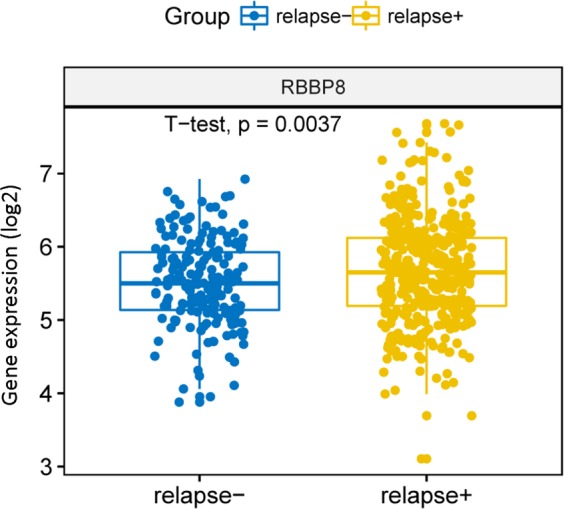
The relationship between the expression of RBBP8 and recurrence, The *X* axis represents no recurrence and recurrence, the *Y* axis represents gene expression. RBBP8 gene expression was measured as log2, *P* = 0.0037, unpaired *t* test, two sided

To investigate the relationship between the RBBP8 expression and the recurrence rate in PCM patients. We analyzed 585 cases PCM patients in GSE83503 data set. This data showed that RBBP8 expression was higher in relapsed patients compared with PCM patients who do not relapse (Fig. 2, *P* = 0.0037, Unpaired *t* test, two sided). The data showed that the high initial expression level of RBBP8 determines the likelihood of recurrence.

**Fig. 3:**
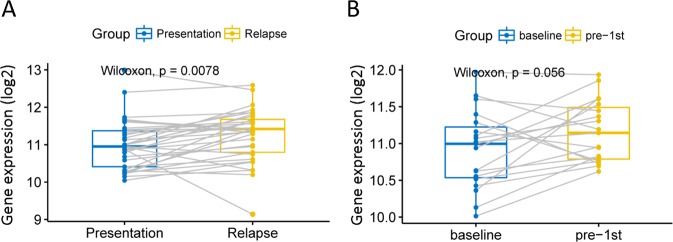
Comparison of RBBP8 expression levels before and after recurrence in the same patient. **a** RBBP8 expression were significantly increase after relapse. The *X* axis represents before and after recurrence and the *Y* axis represents gene expression. RBBP8 gene expression was measured as log2. *P* = 0.0078, paired *t* test, two sided. **b** RBBP8 expression were increase between baseline (diagnosis) and pre-1st (after induction chemotherapy). The *X* axis represents before and after treatment and the *Y* axis represents gene expression (log2), *P* = 0.056, paired *t* test, two sided

To explored the relationship of RBBP8 expression between before and after recurrence in PCM. We asked 33 cases PCM patients with RBBP8 expression from data set GSE82307. RBBP8 expression significantly increases after relapse when compared with pre-relapse (Fig. 3a, *P* = 0.0078, paired *t* test, two sided). We also described 19 cases PCM patients compared with the RBBP8 expression between baseline (diagnosis) and pre-1st (after induction chemotherapy) from the GSE19554 data set. There is an increasing trend between baseline and pre-1st (Fig. 3b, *P* = 0.056, paired *t* test, two sided).

### Expression of RBBP8 genes predicted worse survival in PCM

**Fig. 4:**
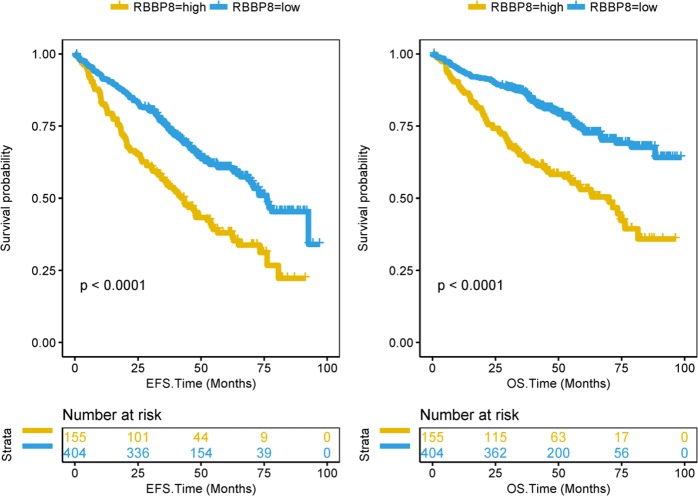
RBBP8 was a prognostic factor in PCM. The *X* axis represents time (month) and the *Y* axis represents survival probability. Kaplan–Meier survival curves show that RBBP8 high expression predict poor event-free survival time (EFS) in PCM (Left plot), *P* < 0.0001, log-rank test. Kaplan–Meier survival curves show that RBBP8 high expression predict poor overall survival (OS) in PCM (Right plot), *P* < 0.0001, log-rank test

We studied the prognosis of PCM patients from the GSE24080 data set. The RBBP8 expression was highly correlated with the EFS and OS of PCM (Fig. 4, EFS, *P* < 0.0001; OS, *P* < 0.0001, log-rank test). The RBBP8-high group was associated with poor survival in PCM, whereas the RBBP8-low group had a good survival (Fig. 4). In addition, Cox regression analysis was performed to verify whether RBBP8 is an independent of clinical prognostic factor for PCM. In GSE24080 data set, it showed that RBBP8 ( > 11.08), B2M ( ≥ 3.5 mg/l), MRI ( ≥ 3 focal lesions) and BMPC ( ≥ 35%) were significantly associated with EFS (Table S1, *P* = 1.28e-03, *P* = 3.54e-02, *P* = 2.40e-02, *P* = 1.74e-02); and RBBP8, B2M, MRI were significantly associated with OS (Table S1, *P* = 1.31e-04, *P* = 1.81e-02, *P* = 9.72e-04). The hazard ratio of RBBP8 ( > 11.08 vs ≤ 11.08) in EFS was 1.57 (95% CI, 1.19–2.06), and the hazard ratio in OS was 1.87 (95% CI, 1.36–2.57). Between RBBP8-high group and RBBP8-low group, there was no significant in baseline patient characteristics such as age, sex, race, and isotype (Table S2, *P* > 0.05, Fisher exact test). Most of clinical characteristic were significantly different especially CRP, LDH, and MRI between these two groups (Table S2, *P* < 0.001, unpaired *t* test, two sided).

### The relationship between RBBP8 and cell cycle in PCM

**Fig. 5:**
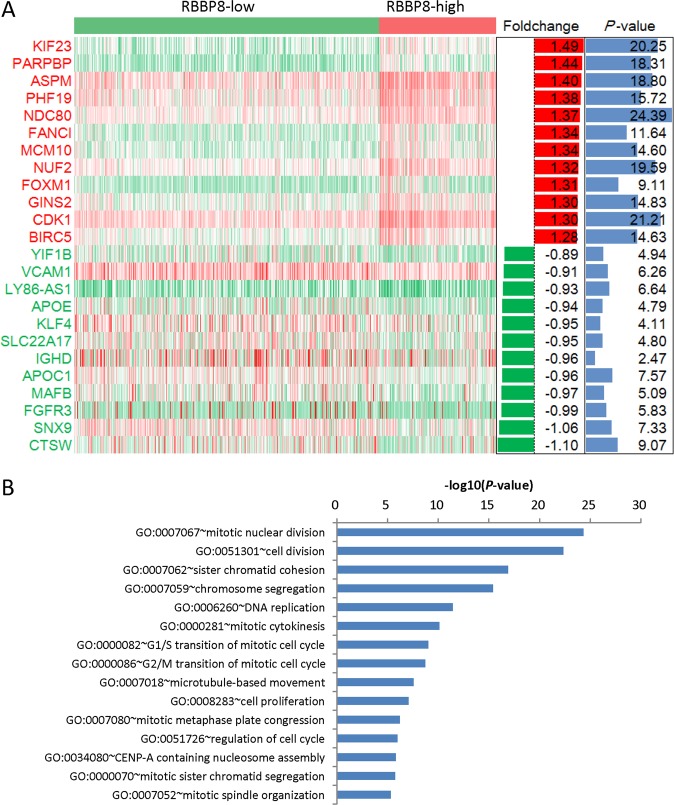
RBBP8 was closely related to cell cycle in PCM. **a** Heat map showed that different expression genes between the RBBP8-high group (red) and RBBP8-low group (green). Top 12 upregulated genes (red) and top 12 downregulated genes (green) were showed. The two bar plots are foldchange (log2, left) and *P* value (−log10, right), respectively. **b** GO analysis of the top 15 pathways showed that RBBP8 was mostly involved DNA replication, cell division, and mitotic nuclear division. *P* value (−log10)

**Fig. 6:**
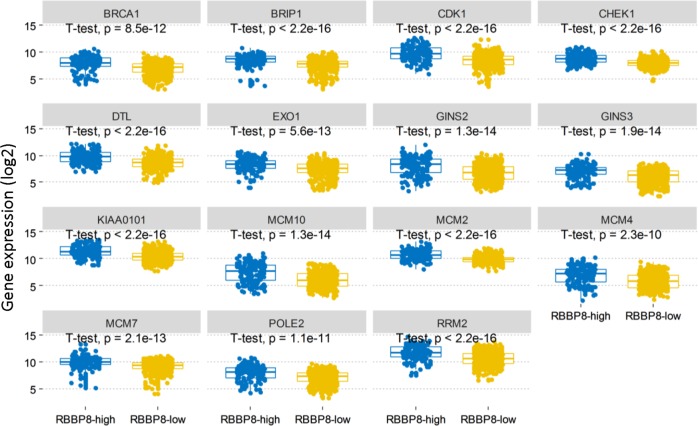
Differentially expressed genes in the DNA replication pathway were displayed. All genes are upregulated. BRCA1, *P* = 8.5e-12; BRIP, CDK1, CHEK1, DHL, KIAA0101, MCM2, RRM2, *P* < 2.2e-16; GINS2, MCM10, *P* = 1.3e-14, EXO1, *P* = 5.6e-13, G1NS3, *P* = 1.9e-14, MCM4, *P* = 2.3e-10, MCM7, *P* = 2.1e-13, POLE2, *P* = 1.1e-11, unpaired *t* test, two sided

To investigate the biological role of RBBP8 expression in PCM, we selected the genes most relevant to RBBP8 for analysis. We found that RBBP8 was positively related to most cell cycle related genes and negatively related to a few cell cycles related genes. Among the 186 genes, 142 genes were upregulated with RBBP8 expression, and 44 genes were downregulated with RBBP8 expression. The heat map showed that top 12 upregulated genes and top 12 downregulated genes correlated with RBBP8 (Fig. 5a, *P* < 0.05). To further elucidate the biologic role of RBBP8 in PCM, we performed GO analysis and showed the top 15 genes. The most significantly enriched pathway for all different expressed genes is DNA replication, cell division, and especially mitotic nuclear division (Fig. 5b, *P* < 0.05). In DNA replication pathway, we showed that 15 different expressed genes (most of these genes are involved in cell division) are upregulated (Fig. 6, *P* < 0.0001, unpaired *t* test, two sided).

### Expression of RBBP8 in therapeutic response of PCM patients

We analyzed RBBP8 expression and clinical treatment response in 238 PCM patients from data set GSE9782. The expression levels of RBBP8 was no significant difference in each treatment response group of biortezomib or Dex (Fig S3, Bortezomib, *P* = 0.37; Dex, *P* = 0.53, ANOVA test). We analyzed RBBP8 expression and clinical response of another 136 PCM patients from data set GSE39754. Similarly, there was no significant difference in the RBBP8 expression among all groups (Fig. S4, *P* = 0.26, ANOVA test).

## Discussion

Mutations in the RBBP8 gene cause tumors such as colorectal cancer and endometrial cancer [29, 30]. Low RBBP8 expression in many types of tumors (such as bladder cancer, ovarian cancer, and breast cancer) has a worse prognosis [21–23]. Presumably, the gene itself may be a tumor suppressor. However, little is known about the prognostic and biological significance of RBBP8 in PCM. We described the expression of RBBP8 in the gene expression profile of 1930 PCM samples (1878 PCM patients) from seven independent datasets, showing that the high RBBP8 expression predicts poorer survival level and relapse and affects the cell cycle in PCM.

PCM is a malignant tumor of terminally differentiated plasma cells. The survival time of PCM patients ranges from a few weeks to > 10 years. As PCM is incurable, the significant differences in the survival of PCM, accurate stratification of patients with prognosis is essential to improve patient outcomes [31, 32]. Patients of PCM who predict of Monoclonal Gammopathy of Undetermined significance (MGUS) had better survival time [33]. Whole-body magnetic resonance imaging was used as a risk stratification tool for asymptomatic PCM [34]. In our analysis, RBBP8 expression levels were significantly associated with EFS and OS in PCM. High expression of RBBP8 predicts worse prognosis. Like B2M and MRI, RBBP8 can be considered as independent prognostic factor for PCM. In different serotypes or molecular typing in PCM, RBBP8 expression was different. This indicates that detecting RBBP8 expression levels in the PCM patients can be predicted the ISS stage in certain serotypes and can predict the prognosis in certain molecular typing.

RBBP8 is a protein involved in transcription, DNA replication, DNA repair, and a key checkpoint of G_1_-phase and G_2_-phase in cell cycle. We found that RBBP8 played an important role in the cell cycle. In particular, we found that many genes involved in the cell cycle are upregulated in the DNA replication pathway. Component of the BRCA1–RBBP8 complex regulates CHEK1 activation and controls the cell cycle G2/M checkpoint for DNA damage [35–37]. RBBP8 identified as an candidate oncogene participated in regulating cell cycle [38]. This function of RBBP8 was also observed in other tumors [16, 19, 39]. DNA replication and cell proliferation may be the cause of progression in PCM patients. As revealed above, RBBP8 played a significant role in the cell cycle in PCM. Therefore, the RBBP8 gene may predict poor PCM survival levels by regulating DNA replication pathways. In the future, relevant genes affecting these pathways should be in-depth studied.

PCM is a disease with a high recurrence rate. Researcher has clarified the potential causes of PCM relapse in the previous [40]. Here, we found the relationship between RBBP8 gene expression and recurrence. This finding indicated that the RBBP8 expression is positively correlated with relapse in PCM. It showed that the high initial expression level of the RBBP8 gene determines the likelihood of recurrence. These results suggested that high expression of RBBP8 can predict more likely to relapse in PCM patients.

However, further research is needed to study the molecular mechanisms of RBBP8 in the development of PCM. Such as, conduct some experimental studies to further verify the results. This time we only described a single gene expression biomarker, and future studies can be combined with multiple gene expression biomarkers to assess the prognosis of PCM.

In summary, high expression of RBBP8 gene predicts worse prognosis in PCM patients. RBBP8 may play an important role in the cell cycle, especially in DNA replication in PCM. High expression of RBBP8 predicts higher relapse rate of PCM. RBBP8 can be considered as a new biomarker for PCM prognosis.

## Supplementary information

Supplementary table

Supplementary Figure
