# Supplementary material for: Prognosis value of RBBP8 expression in plasma cell myeloma
Source: Cancer Gene Ther. 2019 Jan 9;27(1):22–9. doi: 10.1038/s41417-018-0069-3 (PMC7027984; doi:10.1038/s41417-018-0069-3)
Supplement: Supplementary file 1 — Supplementary table [file 41417_2018_69_MOESM1_ESM.docx]

**Figure S1.** Expression of the RBBP8 gene in PCM. The X axis represents the ISS stage and the Y axis represents gene expression. RBBP8 gene expression was measured as log2. A, RBBP8 expression levels were significantly increased in ISS stage II and stage III PCM in GSE24080 dataset, P = 0.0012, Kruskal-Wallis test. B, RBBP8 expression pattern in different serotypes. FLC, P = 0.16, IgA, P = 0.17; IgG, P = 6.6e-05, Kruskal-Wallis test.

**Figure 2.** Comparison of RBBP8 expression levels of 9 molecular subtypes in PCM from GSE19784 dataset. The X axis represents different molecular subtypes and the Y axis represents gene expression. RBBP8 gene expression was measured as log2. P < 2.2e-16, Anova test, ns, *, ** and *** indicate P > 0.05, P <= 0.05, P <= 0.01 and P <= 0.001, respectively. The average of the entire data set is used as a reference group. Each group level is compared to a reference group. Add a horizontal dashed line at the average of reference group.

**Figure S3.** Expression of RBBP8 in bortezomib or Dex therapeutic response of PCM patients. The X axis represents the treatment response group: Complete response (CR), Partial response (PR), minimal response (MR), no change (NC) and progressive disease (PD). the Y axis represents gene expression. RBBP8 gene expression was measured as log2. Bortezomib, P = 0.37; Dex, P = 0.53, Anova test. ns indicate P > 0.05. The average of the entire data set is used as a reference group. Each group level is compared to a reference group. Add a horizontal dashed line at the average of reference group.

**Figure S4.** Expression of RBBP8 in different therapeutic response. 136 PCM patients from dataset GSE39754 were treated with Vincristine, Adriamycin, and Dexamethasone (VAD) induction chemotherapy and Autologous Stem Cell Transplant (ASCT). The X axis represents the therapeutic response: Complete response (CR); Very Good Partial Response (VGPR); Partial Response (PR); No Response, Stable disease (NR); No Response, Progressive disease (Prog). The Y axis represents gene expression. RBBP8 gene expression was measured as log2. P = 0.26, Anova test. The average of the entire data set is used as a reference group. Each group level is compared to a reference group. Add a horizontal dashed line at the average of reference group.

Supplementary table 1. Cox regression analysis of 559 cases of plasma cell myeloma patients with RBBP8 expression as an independent prognostic factor.

|  |  | 95% CI for HR | |  |
| --- | --- | --- | --- | --- |
|  | HR | Lower | Upper | *P*-value |
| EFS |  |  |  |  |
| B2M (>= 3.5 mg/l) | 1.39 | 1.02 | 1.90 | 3.54E-02 |
| ALB (>= 35 g/l) | 0.84 | 0.59 | 1.19 | 3.21E-01 |
| HGB (>= 100 g/l) | 0.80 | 0.59 | 1.08 | 1.41E-01 |
| MRI (>= 3 focal lesions) | 1.37 | 1.04 | 1.80 | 2.40E-02 |
| BMPC (>= 35%) | 1.47 | 1.07 | 2.02 | 1.74E-02 |
| RBBP8 (>11.08) | 1.57 | 1.19 | 2.06 | 1.28E-03 |
|  |  |  |  |  |
| OS |  |  |  |  |
| B2M (>= 3.5 mg/l) | 1.58 | 1.08 | 2.32 | 1.81E-02 |
| ALB (>= 35 g/l) | 0.69 | 0.47 | 1.03 | 6.59E-02 |
| HGB (>= 100 g/l) | 0.89 | 0.62 | 1.27 | 5.06E-01 |
| MRI (>= 3 focal lesions) | 1.78 | 1.26 | 2.51 | 9.72E-04 |
| BMPC (>= 35%) | 1.40 | 0.95 | 2.08 | 9.32E-02 |
| RBBP8 (>11.08) | 1.87 | 1.36 | 2.57 | 1.31E-04 |

ALB, Albumin, g/l; B2M, Beta-2 microglobulin, mg/l; BMPC, Bone marrow biopsy plasma cells (%); HGB, Haemoglobin, g/l; MRI, Number of magnetic resonance imaging (MRI)-defined focal lesions (spine, skull, pelvis); EFS, Event-free survival time (months), defined from date of registration to the occurrence of disease progression or relapse, censored at the date of last contact, or death from any cause; OS, Overall survival time (months), defined from date of censored at the date of last contact or registration to the date of death from any cause. HR, hazard ratio; CI, confidence interval.

Supplementary table 2. Baseline patient characteristics based on the expression level of RBBP8.

|  |  | RBBP8-low | RBBP8-high | *P*-value |
| --- | --- | --- | --- | --- |
| n |  | 404 | 155 |  |
| AGE (mean (sd)) |  | 57.04 (9.79) | 57.54 (8.58) | 0.575 |
| SEX (%) | female | 158 (39.1) | 64 (41.3) | 0.707 |
|  | male | 246 (60.9) | 91 (58.7) |  |
| RACE (%) | other | 49 (12.1) | 13 ( 8.4) | 0.267 |
|  | white | 355 (87.9) | 142 (91.6) |  |
| ISOTYPE (%) | FLC | 52 (13.4) | 32 (21.2) | 0.25 |
|  | IgA | 100 (25.8) | 33 (21.9) |  |
|  | IgD | 2 ( 0.5) | 1 ( 0.7) |  |
|  | IgG | 230 (59.3) | 83 (55.0) |  |
|  | Nonsecretory | 4 ( 1.0) | 2 ( 1.3) |  |
| B2M (mean (sd)) |  | 4.32 (4.98) | 5.80 (6.17) | 0.004 |
| CRP (mean (sd)) |  | 9.27 (17.00) | 17.78 (33.26) | <0.001 |
| CREAT (mean (sd)) |  | 1.23 (1.09) | 1.56 (1.64) | 0.007 |
| LDH (mean (sd)) |  | 162.23 (54.48) | 197.39 (84.17) | <0.001 |
| ALB (mean (sd)) |  | 4.09 (0.56) | 3.95 (0.63) | 0.013 |
| HGB (mean (sd)) |  | 11.36 (1.82) | 10.97 (1.75) | 0.02 |
| ASPC (mean (sd)) |  | 42.05 (23.79) | 44.36 (25.73) | 0.33 |
| BMPC (mean (sd)) |  | 44.96 (25.45) | 50.12 (28.07) | 0.041 |
| MRI (mean (sd)) |  | 9.73 (13.75) | 14.44 (15.96) | 0.001 |

n, number of patients; ASPC, Aspirate plasma cells (%);CRP, C-reactive protein, mg/l; CREAT, Creatinine, mg/dl. B2M, ALB, HGB, MRI, BMPC, RBBP8 statistical methods using unpaired t test, two sided. age, sex, race, and isotype statistical methods using Fisher exact test.
