# Supplementary figures and images for: Prognosis value of RBBP8 expression in plasma cell myeloma
Source: Cancer Gene Ther. 2019 Jan 9;27(1):22–9. doi: 10.1038/s41417-018-0069-3 (PMC7027984; doi:10.1038/s41417-018-0069-3)

Supplementary Figure 1

A

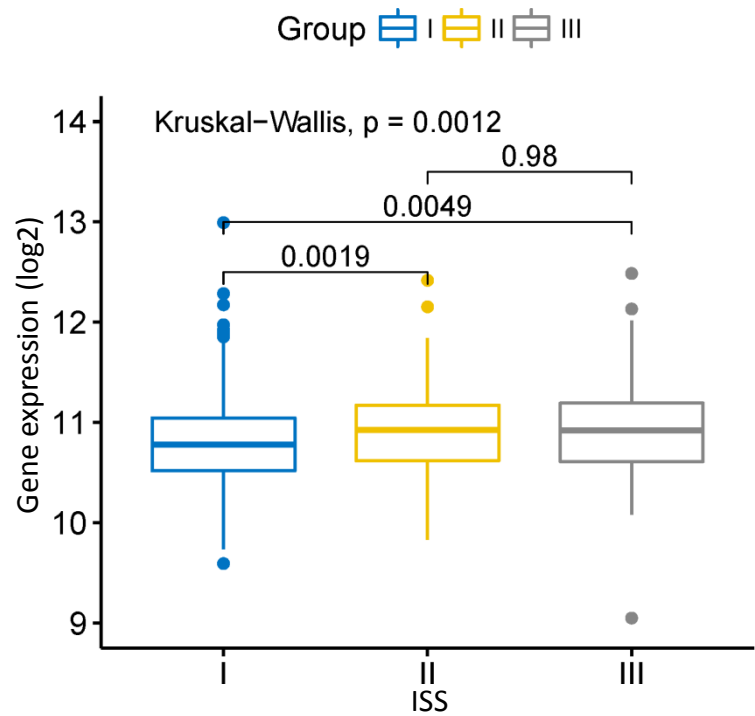

B

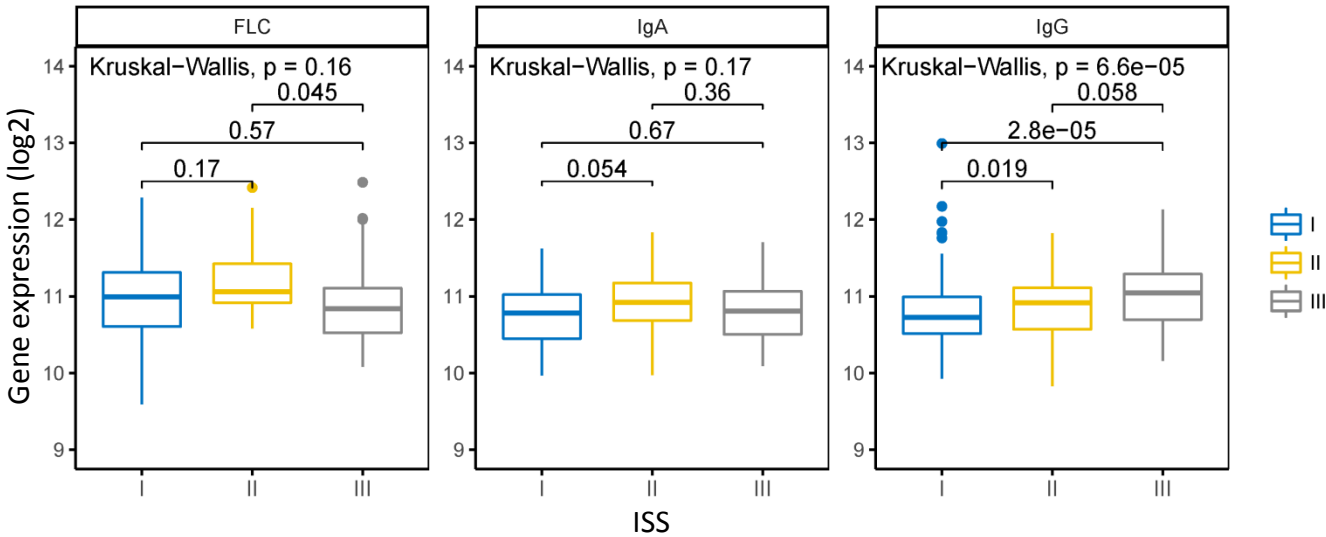

Supplementary Figure 2

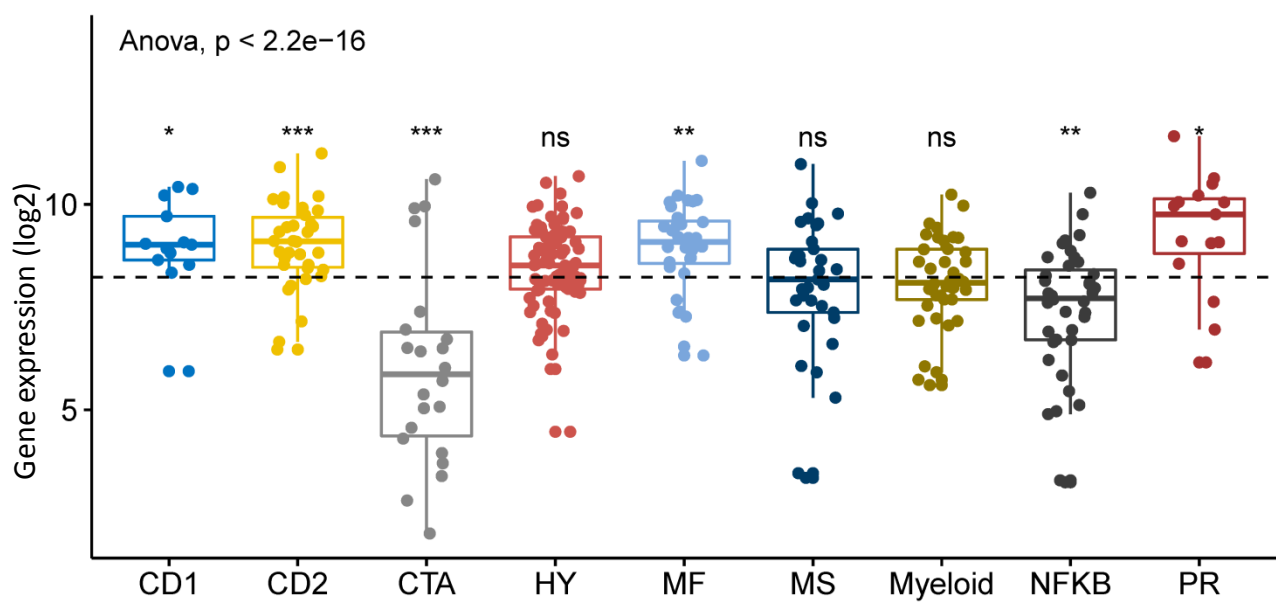

Supplementary Figure 3

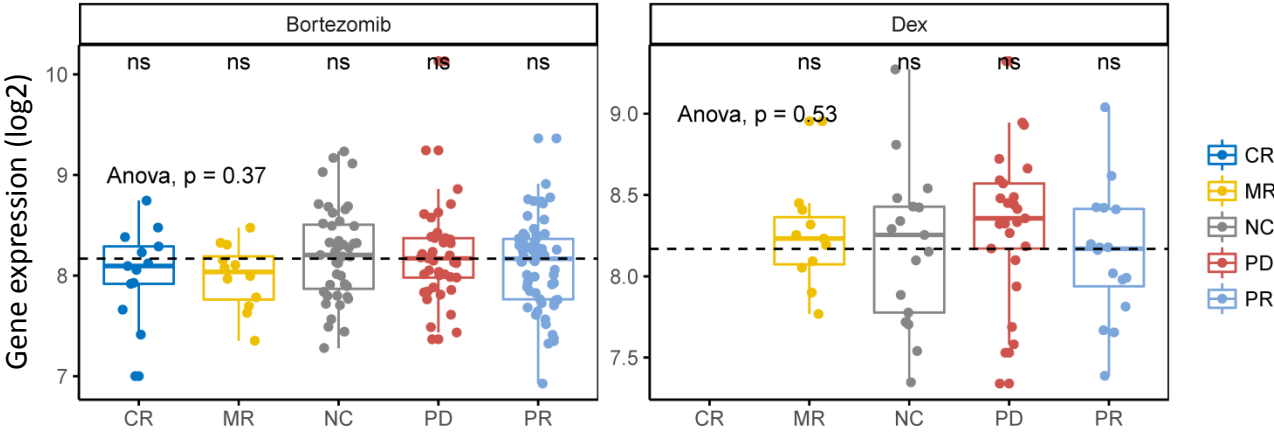

Supplementary Figure 4

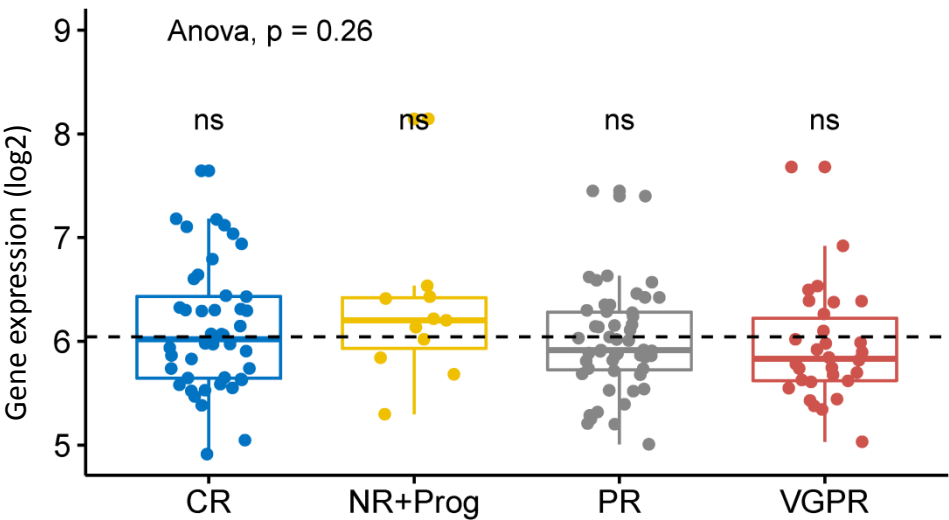

Supplement: Supplementary file 2 — Supplementary Figure [file 41417_2018_69_MOESM2_ESM.pdf]
